# The Influence of Childhood Trauma on the Real‐World Effectiveness of Ketamine in Adults With Treatment‐Resistant Depression

**DOI:** 10.1111/acps.13812

**Published:** 2025-04-16

**Authors:** Danica E. Johnson, Nelson B. Rodrigues, Rodrigo B. Mansur, Roger S. McIntyre, Joshua D. Rosenblat

**Affiliations:** ^1^ Institute of Medical Science, Temerty Faculty of Medicine University of Toronto Toronto Ontario Canada; ^2^ Mood Disorders Psychopharmacology Unit, Poul Hansen Family Depression Centre Toronto Western Hospital—University Health Network Toronto Ontario Canada; ^3^ Department of Psychology, Neuropsychology Track University of Windsor Windsor Ontario Canada; ^4^ Department of Psychiatry University of Toronto Toronto Ontario Canada; ^5^ Department of Pharmacology University of Toronto Toronto Ontario Canada

**Keywords:** childhood trauma, community‐based, ketamine, real‐world effectiveness, treatment‐resistant depression

## Abstract

**Introduction:**

Childhood trauma is a well‐established risk factor for major depressive disorder (MDD) and is often associated with attenuated response to conventional antidepressant therapies. Ketamine has emerged as an effective treatment for treatment‐resistant depression (TRD), but the impact of childhood trauma on its effectiveness remains unclear. Herein, we aimed to determine whether childhood trauma influences the antidepressant effectiveness of ketamine in TRD.

**Methods:**

A retrospective analysis was performed on data from adults with TRD (*n* = 83) who received four ketamine infusions at a community outpatient clinic. Participants were categorized based on cumulative trauma load (high vs. low) and specific trauma types, assessed by the Childhood Trauma Questionnaire (CTQ). Depressive symptoms were measured using the Quick Inventory of Depressive Symptomatology Self‐Report 16‐item (QIDS‐SR16) at baseline and following each infusion. Linear mixed models and chi‐square tests were used to evaluate the impact of trauma on ketamine's antidepressant effectiveness.

**Results:**

Depressive symptoms significantly decreased across all participants over time, with an average reduction of 5.7 points in QIDS‐SR16 scores (*p* < 0.001). High childhood trauma load was reported by 55% of participants. Response rates were 25% in the high trauma load group and 19% in the low trauma load group, while remission rates were 14% and 11%, respectively. However, there were no significant differences in antidepressant effectiveness (*p* = 0.572), response rates (*p* = 0.230), or remission rates (*p* = 0.397) between participants with high versus low trauma loads. Further analysis also revealed no significant associations between specific types of childhood trauma and antidepressant effectiveness, response, or remission outcomes.

**Conclusion:**

Childhood trauma did not attenuate ketamine's antidepressant effectiveness in TRD. These findings support ketamine as a viable treatment for individuals with TRD, including those with significant trauma histories. Further research is warranted to replicate these findings and explore underlying mechanisms.

**Summary**

- Significant outcomes
  ○ Ketamine significantly reduced depressive symptoms across all participants, regardless of the extent of trauma exposure or perceived severity, with an average QIDS‐SR16 score reduction of 5.7 points.
  ○ Neither specific types of trauma (e.g., physical abuse, sexual abuse) nor cumulative trauma severity significantly impacted depressive symptom reduction or remission outcomes following ketamine treatment.
  ○ These findings suggest that ketamine is a viable treatment option for TRD, even among individuals burdened by significant childhood trauma, highlighting its potential to address gaps in traditional antidepressant efficacy.
- Limitations
  ○ The retrospective study design, reliance on self‐reported trauma histories, and absence of a placebo control group introduce potential biases, including recall bias, expectancy effects, and challenges in isolating ketamine's true antidepressant effects.
  ○ The relatively small sample size limited the study's statistical power to detect smaller effects and examine the influence of multiple baseline characteristics comprehensively.
  ○ The use of the ECHO‐wide Cohort version of the Childhood Trauma Questionnaire (CTQ), which includes only six yes/no items, limited our ability to quantify trauma severity or frequency and reduced comparability with studies using the original CTQ [1], which provides subscale scores and clinical cut‐offs.

## 1 Introduction

Major depressive disorder (MDD) is a complex and debilitating mental health condition with a multifactorial etiology, including both genetic and environmental influences [2]. Among the most potent environmental risk factors for the development of MDD is childhood trauma [3, 4, 5, 6]. Research has consistently demonstrated a strong association between childhood trauma and the onset, symptomatology, and course of depressive symptoms in adulthood [7, 8]. Meta‐analytic evidence has shown that experiencing any form of maltreatment during childhood is associated with a more than twofold increase in the risk of developing depression in later life [9]. Moreover, individuals with a history of childhood trauma are more likely to develop chronic or treatment‐resistant depression (TRD), characterized by a more prolonged and severe course of illness that is less responsive to most, but not all (i.e., vortioxetine [10]) conventional antidepressant therapies [8, 11, 12, 13, 14, 15, 16, 17].

The mechanisms underlying this relationship are complex and likely involve both psychosocial and neurobiological factors. Psychosocial factors such as maladaptive emotion regulation strategies [18] and cognitive vulnerabilities, including self‐criticism and heightened emotional reactivity [19], have been implicated in mediating the link between childhood trauma and depression. Neurobiologically, childhood trauma has been associated with lasting changes in brain network connectivity, particularly, in regions involved in emotion regulation and cognitive control, altered neuroendocrine responses (i.e., increased inflammation), and dysregulation of the hypothalamic–pituitary–adrenal (HPA) axis [3, 6]. These alterations may predispose individuals to greater vulnerability to depressive episodes in response to stress later in life and may also contribute to the reduced efficacy of standard antidepressant treatments seen in patients with significant childhood trauma. In addition to the aforementioned changes in brain structure and functional connectivity, the biosignature of adults with MDD reporting trauma is dissimilar from those not reporting trauma, with a much higher likelihood of traumatized adults exhibiting a pro‐inflammatory imbalance, insulin resistance, and cortisol dysregulation [20, 21, 22, 23]. These findings highlight the need for alternative therapeutic approaches that can effectively address the unique challenges faced by this population.

Ketamine, an *N*‐methyl‐d‐aspartate (NMDA) receptor antagonist, has been established as an efficacious treatment for TRD, offering rapid and sustained antidepressant effects in patients who have not responded to traditional therapies [24, 25, 26]. Recent studies have begun to explore the relationship between childhood trauma and ketamine's therapeutic effect, with mixed results [27, 28, 29].

In the first study to explore this connection, O'Brien et al. found that individuals with a higher burden of childhood trauma exhibited a more favorable response to ketamine treatment [27]. Specifically, a significant correlation was observed between the severity of maltreatment and reductions in depressive symptoms following repeated ketamine infusions. Patients with higher scores in areas such as physical neglect and sexual abuse experienced greater reductions in depressive symptoms compared to those with lower scores in these domains.

In a subsequent study, O'Brien et al. identified two groups of patients with similar baseline depression severity but differing response trajectories to repeated IV ketamine [28]. The key differentiating factor between these groups was their exposure to childhood physical abuse. The group that showed a modest (21%) improvement in depressive symptomatology following ketamine treatment had no history of childhood physical abuse, while the group that demonstrated a substantial (61%) improvement did have such a history.

However, in a third study, the same research group failed to replicate these findings [29]. While they were able to identify two groups of patients with similar baseline depression severity and differing response trajectories to repeated IV ketamine, a history of childhood physical abuse did not predict better outcomes [29]. This suggests that factors beyond childhood trauma, such as clinical characteristics, demographic variables, or baseline symptom profiles, may have influenced treatment outcomes. These findings raise the possibility that childhood trauma alone may not be a primary determinant of ketamine response, but rather one of many interacting factors contributing to individual variability in treatment efficacy [29].

Therefore, while childhood trauma is a well‐documented contributor to treatment resistance with conventional monoamine‐based antidepressants [8, 15, 16, 30], the extent to which childhood trauma uniquely influences ketamine's antidepressant response remains uncertain. To address this gap, this study aimed to evaluate whether childhood trauma load or specific trauma types influence the antidepressant effectiveness of IV ketamine in individuals with TRD. By examining both cumulative trauma exposure and self‐reported trauma severity, we sought to clarify whether trauma‐exposed individuals exhibit a distinct treatment response. We hypothesized that individuals with higher trauma load (i.e., a greater number of endorsed trauma types) or perceived trauma severity would demonstrate a commensurate anti‐depressive response to IV ketamine compared to individuals with lower trauma load or severity. Given the dearth of reliable treatment options for this population, the results of this study may inform the development of more personalized and effective interventions for individuals with TRD and a history of childhood trauma.

## 2 Materials and Methods

### 2.1 Study Design and Participants

We performed a retrospective post hoc analysis of data from patients referred to the Canadian Rapid Treatment Center of Excellence (CRTCE), a community outpatient ketamine clinic in Mississauga, Ontario, Canada. As previously outlined [31], patients aged 18–65 with a primary diagnosis of TRD (i.e., inadequate response to at least two different major classes of antidepressants at a sufficient length and dosage) [32] underwent a series of four IV ketamine hydrochloride infusions over a span of 8–14 days. The first two infusions were administered at a dose of 0.5 mg/kg (diluted in 0.9% saline solution and infused over 40 min), while the subsequent two infusions were flexibly dosed between 0.5 and 0.75 mg/kg, based on clinical response and tolerability.

Upon completing the four infusions, patients were invited to participate in a pharmacogenetic add‐on study conducted from January 2021 to July 2021. This study was approved by a community institutional research ethics board and registered under the identifier NCT04695405. Due to the COVID‐19 pandemic, all study procedures were carried out remotely. Eligibility criteria required participants to have a prior diagnosis of MDD, be aged 18 to 65, have undergone at least four ketamine infusions at the CRTCE for TRD, and have no history of substance use disorder, alcohol use disorder, or psychosis. After providing consent, participants were sent a genetics kit containing saliva swabs from Inagene Diagnostics Inc. and completed additional study scales via telephone, including the CTQ.

### 2.2 Data Collection

We collected sociodemographic and clinical data retrospectively as part of the pharmacogenetic add‐on study. This included information on sex, age, race, educational attainment, age of first major depressive episode (MDE), and whether they had insurance coverage for the ketamine treatments. Participants completed the Quick Inventory of Depressive Symptomatology Self‐Report 16‐item (QIDS‐SR16) [33] at baseline, before the second, third, and fourth infusions, and during a posttreatment visit after the fourth infusion. These assessments occurred an average of 2.4 days apart to track the impact of ketamine treatment [31]. Due to the real‐world nature of the analysis, 25 patients did not complete all four post‐infusion assessments, resulting in missing QIDS‐SR16 scores for some time points.

We utilized the “Environmental Influences on Child Health Outcomes (ECHO)‐wide Cohort” version of the CTQ [34], which includes six yes/no questions on topics such as the death of a close friend or family member, major upheaval between parents, traumatic sexual experience, victim of violence, severe illness or injury, and other major upheavals before age 18. This version allowed for a maximum total score of six and a minimum score of zero.

To examine the impact of childhood trauma on ketamine's antidepressant effectiveness, we categorized participants into high or low trauma load groups based on CTQ scores. The “High Load” group included individuals with scores between 3 and 6 or who reported experiencing sexual trauma (CTQ Question 3) or violence (CTQ Question 4). This classification aligns with evidence that higher trauma loads, particularly, those involving abuse (i.e., physical, sexual, and emotional), are associated with poorer treatment outcomes and greater chronicity in depression [8, 15, 16, 30]. The “Low Load” group comprised individuals with scores from 0 to 2, reflecting exposure to fewer distinct trauma categories rather than an absence of significant trauma.

This classification does not capture repeated exposure to a single category of trauma (e.g., chronic illness or multiple family deaths), which could still have a profound impact on mental health. Therefore, we utilized the self‐reported severity scores to supplement this analysis. Participants who endorsed experiencing any of the six trauma types rated the severity of each trauma on a 7‐point Likert scale (1 = *Not at All Traumatic* to 7 = *Extremely Traumatic*). We calculated a mean trauma severity score for each participant by averaging the ratings for all endorsed trauma types. We also computed a total trauma severity score by summing the Likert scale ratings across all endorsed trauma types. These continuous measures complemented the dichotomous trauma load classification, providing insight into subjective trauma severity from both an averaged and cumulative perspective.

Finally, to analyze the impact of specific types of childhood trauma on ketamine's effectiveness, we categorized participants based on their responses to individual CTQ items. Those who answered “yes” to a trauma type were classified as having experienced that trauma, while those who answered “no” were classified as not having experienced it.

### 2.3 Statistical Analysis

We conducted all statistical analyses using SPSS software, version 29.0.1.0 (SPSS Inc., Chicago, IL, USA). Two separate repeated‐measures linear mixed models (LMM) were performed to explore the effect of childhood trauma on ketamine's effectiveness for depressive symptoms across multiple time points. For each model, we checked assumptions thoroughly. Scatterplots of residuals versus fitted values verified linearity and homoscedasticity, while Q‐Q plots assessed normality. We evaluated autocorrelation in residuals using autocorrelation function (ACF) plots.

The first LMM examined the effects of trauma load on depressive symptoms (QIDS‐SR16 scores) at each time point, with fixed effects for trauma load (high vs. low), time (infusion number), and their interaction. We included participant ID as a random effect to account for repeated measures. The second LMM evaluated the effects of specific trauma types on antidepressant outcomes, with each trauma type entered as a separate predictor. Both models used a first‐order autoregressive (AR1) covariance structure based on Akaike and Bayesian Information Criteria (AIC and BIC). To control for Type I error due to conducting two LMMs with the same dependent variable (QIDS‐SR16 scores), we set the alpha level to 0.025 for the main effects and interaction terms in each model. For post hoc pairwise comparisons within each LMM, we applied a Bonferroni correction to account for the multiple comparisons. Adjusted *p*‐values were then compared against an alpha level of 0.05 for significance. Effect sizes are reported as partial *η* ^2^. We also conducted Little's MCAR test to assess whether missing QIDS‐SR16 scores were missing completely at random (MCAR). The test was nonsignificant (*χ* ^2^(15) = 22.505, *p* = 0.095), suggesting that missingness was random. Given this, we proceeded with the LMM, which assumes data are missing at random (MAR) and is robust to missing data.

To supplement these analyses, we conducted Pearson's correlations to examine whether self‐reported childhood trauma severity was associated with changes in depressive symptoms following ketamine treatment. Specifically, we assessed the relationship between (1) average trauma severity (mean Likert scale score across endorsed trauma types) and (2) cumulative trauma severity (sum of Likert scale scores across all trauma types) with change in QIDS‐SR16 scores (baseline minus postinfusion 4). Scatterplots verified linearity, and Shapiro–Wilk tests confirmed normality. Missing postinfusion 4 QIDS‐SR16 scores (*n* = 20) were imputed using Last Observation Carried Forward (LOCF), ensuring all participants had a complete dataset for the change score calculation.

We also conducted chi‐square tests to evaluate relationships between trauma load or specific trauma types and response/remission rates. Missing postinfusion four scores (*n* = 20) were imputed using LOCF to complete the analysis. We set the significance threshold at *α* = 0.05 for the overall trauma load analysis and applied a Bonferroni correction (*α* = 0.0083) for the analysis of specific trauma types to account for multiple comparisons.

## 3 Results

### 3.1 Sociodemographic and Clinical Characteristics

A total of 84 participants completed the pharmacogenetic add‐on study. The final sample consisted of 83 participants; 37 participants were classified as Low Load, and 46 were classified as High Load. One participant was excluded from the analysis for lacking baseline QID‐SR16 scores. Participant sex, race, age, highest level of education, insurance status, age of first MDE, and baseline mean QIDS‐SR16, GAD‐7, and CTQ scores are reported in Table 1.

**TABLE 1:** Sociodemographic and clinical characteristics of the Low Load (*n* = 37) and High Load (*n* = 46) subgroups.

Baseline characteristics	Low Load, *n* (%)	High Load, *n* (%)
Sex		
Female	21 (56.8)	34 (73.9)
Male	16 (43.2)	12 (26.1)
Race		
White	31 (83.8)	42 (91.3)
Indigenous	0 (0)	1 (2.2)
Latin American	1 (2.7)	2 (4.3)
Middle Eastern	1 (2.7)	0 (0)
South Asian	2 (5.4)	1 (2.1)
Southeast Asian	2 (5.4)	0 (0)
Highest level of education		
< High school	1 (2.7)	1 (2.2)
High school graduate	6 (16.2)	3 (6.5)
Some college/university	12 (32.4)	15 (32.6)
Associate's degree	1 (2.7)	2 (4.3)
Bachelor's degree	11 (29.7)	12 (26.1)
Graduate degree	1 (2.7)	8 (17.4)
Professional degree	5 (13.5)	5 (10.9)
Insurance coverage		
No	31 (83.8)	34 (73.9)
Yes	6 (16.2)	12 (26.1)
Mean age (SD)	37.5 (13.6)	44.9 (12.4)
Mean age of first MDE	20.9 (11.3)	19.3 (9.5)
Mean baseline QIDS‐SR16 (SD)	17.5 (4.4)	18.3 (4.4)
Mean baseline GAD‐7 (SD)	13.0 (5.5)	13.2 (5.3)
Mean baseline trauma load (SD)	0.9 (0.8)	3.5 (1.2)
Mean baseline average trauma severity (SD)	0.7 (0.8)	3.2 (1.5)
Mean baseline cumulative trauma severity (SD)	4.5 (4.6)	19.1 (8.7)

Abbreviations: CTQ: childhood trauma questionnaire; GAD‐7: generalized anxiety disorder 7‐item; MDE: major depressive episode; QIDS‐SR16: quick inventory of depressive symptomatology‐self‐report; SD: standard deviation.

### 3.2 Effect of Childhood Trauma Load and Severity on Depressive Symptomology

An LMM was conducted to determine if childhood trauma load affects the antidepressant effectiveness of ketamine as measured by the QIDS‐SR16 scores. Scatter plots of the residuals versus fitted values supported the assumptions of linearity and homoscedasticity. Q‐Q plots revealed that the residuals followed the normal distribution closely, supporting the assumption of normality of residuals. The ACF plot of the residuals suggested a minor negative autocorrelation at lag 2, as indicated by the lag 2 coefficient falling slightly outside the lower confidence interval. However, given the small magnitude of this autocorrelation and the absence of significant autocorrelation at other lags, we did not adjust for it in our model.

**FIGURE 1:**
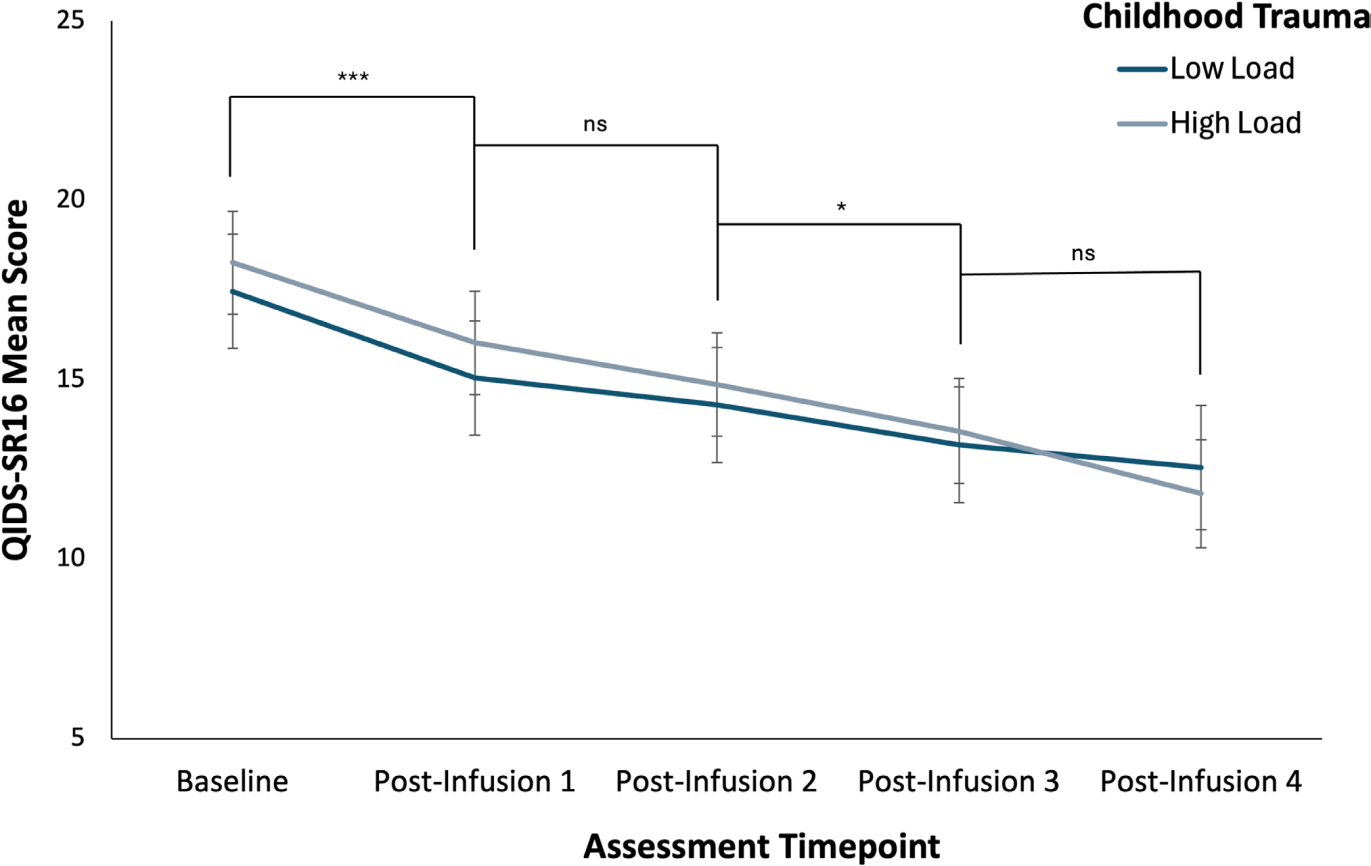
Mean QIDS‐SR16 scores at baseline and after each subsequent infusion for the High Load and Low Load subgroups. Error bars represent 95% confidence intervals. ***p ≤ 0.001, *p ≤ 0.05, ns *p* > 0.05.

The analysis revealed a significant main effect of time on QIDS‐SR16 scores, *F*(4, 183.86) = 32.19, *p* < 0.001, partial *η* ^2^ = 0.41, indicating a significant reduction in depressive symptoms over time (Figure 1). Post hoc pairwise comparisons with Bonferroni correction confirmed significant reductions in QIDS‐SR16 scores from baseline at all subsequent time points (*p* < 0.001, Table 2). Additionally, significant decreases were observed from postinfusions 1–3 (*p* < 0.001) and postinfusion 4 (*p* < 0.001; Table 2). Further decreases were also significant from postinfusions 2–3 (*p* = 0.041; Table 2) and post‐infusions 2–4 (*p* = < 0.001). However, there was no significant change in depressive symptoms between postinfusions 1–2 (*p* = 0.200; Table 2) and postinfusions 3–4 (*p* = 0.108; Table 2).

**TABLE 2:** Post hoc pairwise comparisons of QIDS‐SR16 scores.

Timepoints	Mean difference^a^	SE	*p* ^b^	95% confidence interval	
				Lower bound	Upper bound
Baseline − Infusion 1	−2.320	0.407	**< 0.001**	−3.471	−1.166
Baseline − Infusion 2	−3.277	0.466	**< 0.001**	−4.597	−1.957
Baseline − Infusion 3	−4.484	0.489	**< 0.001**	−5.875	−3.093
Baseline − Infusion 4	−5.672	0.527	**< 0.001**	−7.172	−4.172
Infusion 1 − Infusion 2	−0.959	0.410	0.200	−2.119	0.201
Infusion 1 − Infusion 3	−2.166	0.475	**< 0.001**	−3.510	−0.822
Infusion 1 − Infusion 4	−3.364	0.524	**< 0.001**	−4.842	−1.866
Infusion 2 − Infusion 3	−1.207	0.417	0.**041**	−2.387	−0.027
Infusion 2 − Infusion 4	−2.395	0.508	**< 0.001**	−3.834	−0.956
Infusion 3 − Infusion 4	−1.188	0.463	0.108	−2.499	0.123

*Note*: This table reflects data for the full sample of TRD patients, regardless of their assignment to High or Low Load groups.

^a^ Negative values represent a decrease in QIDS‐SR16 score.

^b^ Bonferroni‐corrected *p* values. Bolded values are significant.

No significant main effects were found for group (*F*(1, 81.87) = 0.19, *p* = 0.668) or group‐by‐time interaction (*F*(4, 183.86) = 0.73, *p* = 0.572), indicating that childhood trauma load did not statistically differentiate the groups in terms of QIDS‐SR16 scores (Figure 1).

Additionally, chi‐square tests were conducted to examine the relationship between trauma load (High vs. Low), response rates (responders vs. nonresponders), and remission rates (remitter vs. nonremitter) following ketamine treatment. Among the 46 patients in the High Load group, 14 (30%) patients met criteria for response (i.e., ≥ 50% reduction in QIDS‐SR16 total score), and 8 (17%) achieved remission (i.e., ≤ 5 points on the QIDS‐SR16). In comparison, 7 (19%) of the 37 patients in the Low Load group met criteria for response (i.e., ≥ 50% reduction in QIDS‐SR16 total score), and 4 (11%) achieved remission (i.e., ≤ 5 points on the QIDS‐SR16). However, the chi‐square tests revealed no statistically significant association between trauma load and treatment response, *χ* ^2^(1, *N* = 83) = 1.44, *p* = 0.230, nor trauma load and remission status, *χ* ^2^(1, *N* = 83) = 0.72, *p* = 0.397.

To further support these results, we conducted two Pearson's correlations to examine the relationship between self‐reported severity of childhood trauma (measured as both average and cumulative Likert scale scores) and change in depressive symptoms following ketamine treatment. Both relationships were linear, with all variables normally distributed, as assessed by Shapiro–Wilk's test (*p* > 0.05), and no outliers were detected. Our analysis revealed no significant correlation between average trauma severity and the change in depressive symptoms (*r*(81) = 0.124, *p* = 0.266), with average trauma severity accounting for only 1.54% of the variance in depressive symptom improvement. Similarly, cumulative trauma severity was not significantly correlated with changes in depressive symptoms (*r*(81) = −0.112, *p* = 0.312), explaining only 1.25% of the variance. These findings suggest that both the load and perceived severity of childhood trauma, whether measured as an average or total severity score, are minimally associated with antidepressant response to ketamine.

### 3.3 Antidepressant Response and Types of Childhood Trauma

A second LMM was conducted to determine if the presence or absence of specific types of childhood trauma affects the antidepressant effectiveness of ketamine as measured by QIDS‐SR16 scores. Each trauma type was entered as a separate predictor in the model. Table 3 presents the frequencies of participants according to their responses to the six CTQ questions. Scatter plots of the residuals versus fitted values supported the assumptions of linearity and homoscedasticity. Q‐Q plots revealed that the residuals followed the normal distribution closely, supporting the assumption of normality of residuals. The ACF plot of the residuals suggested a minor negative autocorrelation at lag 2, as indicated by the lag 2 coefficient falling slightly outside the lower confidence interval. However, given the small magnitude of this autocorrelation and the absence of significant autocorrelation at other lags, we did not adjust for it in our model.

**TABLE 3:** Distribution of responses to Childhood Trauma Questionnaire items.

CTQ Item	Response	Frequency	Percentage
CTQ Question 1: DEATH of close friend/family	No	44	53.0
	Yes	39	47.0
CTQ Question 2: Major upheaval between parents	No	53	63.9
	Yes	30	36.1
CTQ Question 3: Traumatic sexual experience	No	58	69.9
	Yes	25	30.1
CTQ Question 4: Victim of violence	No	54	65.1
	Yes	29	34.9
CTQ Question 5: Extremely ill or injured	No	60	72.3
	Yes	23	27.7
CTQ Question 6: Other significant upheavals	No	35	42.2
	Yes	48	57.8

*Note*: This table presents the frequency and percentage of participants who reported experiencing each of the six childhood trauma types assessed by the CTQ. Trauma types include the death of a close friend or family member, major upheaval between parents, traumatic sexual experience, victim of violence, severe illness or injury, and other significant upheavals. Responses are categorized as “Yes” (trauma experienced) or “No” (trauma not experienced).

**FIGURE 2:**
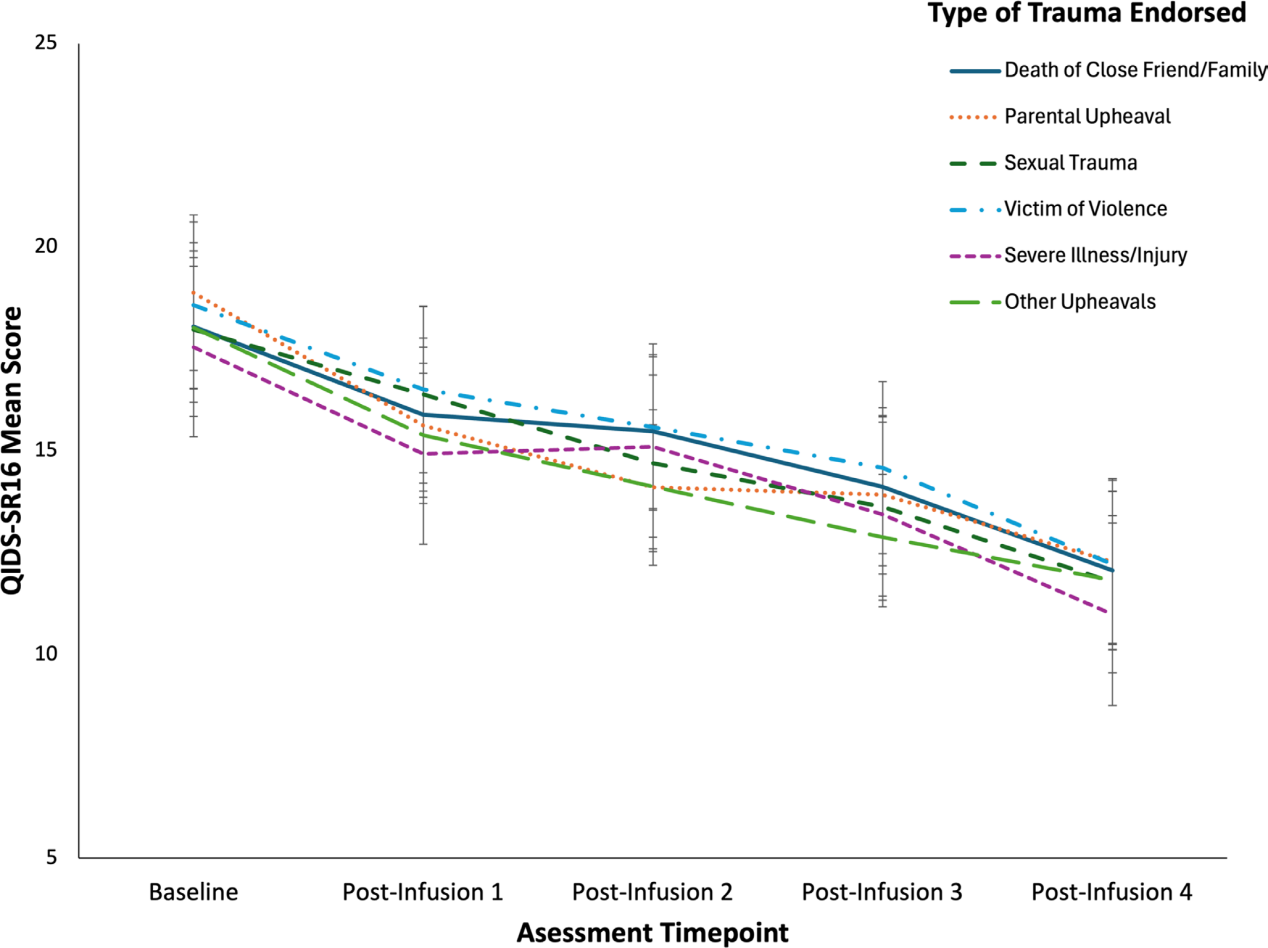
Lines represent the trajectory of depressive symptoms (mean QIDS‐SR16 score), across the five assessment timepoints for each type of trauma experienced, as determined by the CTQ. Error bars represent 95% confidence intervals.

Consistent with the findings from the High Load versus Low Load comparison, the analysis revealed a significant main effect of time on QIDS‐SR16 scores, *F*(4, 171.88) = 23.30, *p* < 0.001, partial *η* ^2^ = 0.35, indicating a significant reduction in depressive symptoms over time (Figure 2). No significant main effects were found for group, indicating that the presence or absence of specific types of childhood trauma did not statistically differentiate the groups in terms of QIDS‐SR16 scores (all *p*‐values > 0.05, Figure 2).

Initially, a significant group‐by‐time interaction was detected for Question 2 on the CTQ (i.e., major upheaval between parents before the age of 18), with *F*(4, 169.58) = 2.60, *p* = 0.038, partial *η* ^2^ = 0.0578 (Figure S1b). However, after applying a Bonferroni correction to account for multiple comparisons across LMMs and adjusting the significance threshold to *α* = 0.025, this interaction no longer reached statistical significance. Similarly, no significant group‐by‐time interactions were found for the other types of childhood trauma assessed by the CTQ (all *p*‐values > 0.05), indicating that the presence or absence of specific types of trauma did not significantly differentiate the groups in terms of changes in QIDS‐SR16 scores over time (Figure S1a,c–f). Effect sizes for group‐by‐time interactions across the remaining CTQ items ranged from small to moderate, with partial *η* ^2^ values between 0.0082 and 0.0393, further supporting that the presence or absence of specific trauma types had minimal impact on changes in QIDS‐SR16 scores over time.

To examine the association between the six different types of childhood trauma identified by the CTQ and response and remission rates following ketamine treatment, chi‐square tests were conducted. The results indicated no significant associations between the presence of specific types of traumas and the likelihood of achieving a response or remission. Detailed chi‐square values and corresponding *p*‐values for each trauma type are summarized in Table 4.

**TABLE 4:** Chi‐square test results for the association between childhood trauma types and ketamine treatment response and remission rates.

CTQ Question	Response rate					Remission rate				
	Trauma type present (%)	Trauma type absent (%)	*χ* ^2^	df	*p*	Trauma type present (%)	Trauma type absent (%)	*χ* ^2^	df	*p*
CTQ Question 1: Death of close friend/family	33.3% (13/39)	18.2% (8/44)	2.511	1	0.113	17.9% (7/39)	11.4% (5/44)	0.725	1	0.395
CTQ Question 2: Major upheaval between parents	33.3% (10/30)	20.8% (11/53)	1.604	1	0.205	16.7% (5/30)	13.2% (7/53)	0.185	1	0.667
CTQ Question 3: Traumatic sexual experience	28.0% (7/25)	24.1% (14/58)	0.138	1	0.710	20.0% (5/25)	12.1% (7/58)	0.889	1	0.346
CTQ Question 4: Victim of violence	31.0% (9/29)	22.2% (12/54)	0.775	1	0.379	17.2% (5/29)	13.0% (7/54)	0.279	1	0.597
CTQ Question 5: Extremely ill or injured	39.1% (9/23)	20.0% (12/60)	3.220	1	0.073	17.4% (4/23)	13.3% (8/60)	0.221	1	0.638
CTQ Question 6: Other significant upheavals	27.1% (13/48)	22.9% (8/35)	0.191	1	0.662	18.8% (9/48)	8.6% (3/35)	1.696	1	0.193

*Note*: This table presents the response and remission rates following ketamine treatment based on the presence or absence of specific types of childhood trauma, as assessed by the CTQ. Response is defined as a ≥ 50% reduction in QIDS‐SR16 total score, and remission is defined as a QIDS‐SR16 score of **≤** 5 after four ketamine infusions. The percentages indicate the proportion of patients who met the criteria for response or remission within each group (trauma type present vs. trauma type absent). The chi‐square test (*χ* ^2^) was used to evaluate the statistical significance of differences between groups, with degrees of freedom (df) and *p*‐values provided for each comparison. A Bonferroni correction was applied to account for six comparisons, setting the significance value at *p* < 0.0083.

## 4 Discussion

The present study aimed to explore the impact of childhood trauma on the antidepressant effectiveness of ketamine in individuals with TRD. Our findings revealed a significant main effect of time, demonstrating that depressive symptoms decreased across all participants throughout the course of treatment. Consistent with our hypothesis, no significant differences were observed in antidepressant effectiveness or response/remission rates between participants with high versus low trauma loads. Additionally, the presence or absence of specific types of childhood trauma did not significantly influence the reduction in depressive symptoms following repeated ketamine infusions. These results suggest that, regardless of the severity or type of childhood trauma, ketamine may be equally effective in reducing depressive symptoms in individuals with TRD. This challenges the prevailing notion that individuals with severe childhood trauma respond less favorably to pharmacological interventions and supports ketamine as a viable treatment option for this population.

Our findings align with prior research documenting ketamine's rapid and robust antidepressant effects in both real‐world effectiveness studies and randomized controlled trials, as highlighted in recent meta‐analyses [24, 26]. In terms of the relationship between ketamine's antidepressant effect and childhood trauma, our findings also align with O'Brien et al.'s most recent replication study, which failed to support that a history of childhood physical abuse predicted better outcomes [29]. Additionally, a recent comprehensive meta‐analysis showed that individuals with childhood trauma benefit similarly from active antidepressant treatments, including pharmacotherapy and psychotherapy, compared to those without trauma [35]. Notably, there was no significant difference in treatment effects between individuals with and without childhood trauma, regardless of trauma type, study design, depression diagnosis, assessment method, study quality, or treatment type and length [35].

However, our results contrast with the earliest study by O'Brien et al. [27], which reported that patients with significant childhood sexual abuse, physical abuse, and cumulative maltreatment load responded better to both single (*n* = 115) and repeated (*n* = 63) ketamine infusions than patients without this history. While our sample size for repeated infusions (*n* = 83) was modestly larger, this difference is unlikely to fully account for the discrepancy in findings. We believe methodological differences, particularly in trauma assessment, may be more influential.

O'Brien et al. [27] used a version of the CTQ that focuses specifically on childhood maltreatment and provides total and subscale scores for five distinct domains: sexual abuse, physical abuse, physical neglect, emotional abuse, and emotional neglect [1]. It also provides recommended cut‐off scores for identifying none, mild, moderate, and severe exposure levels for each type of childhood maltreatment [1].

In contrast, our study employed a version of the CTQ that captures both childhood maltreatment and other adverse experiences, including the loss of a close friend or family member, parental divorce/separation, severe illness or injury, and other major childhood upheavals [34]. While this measure provides insight into a range of childhood adversities that may impact antidepressant response, it does not generate subscale scores or clinical thresholds specific to maltreatment severity. These differences in trauma assessment may help explain the discrepancy between our findings and those of O'Brien et al. [27], where childhood maltreatment was associated with a stronger ketamine response.

One possible explanation is that combining diverse childhood adversities may have diluted the specific effects of maltreatment‐related experiences on antidepressant response. However, this seems unlikely, as 41 of the 46 participants in our High Load group reported exposure to sexual or physical violence before age 18, indicating that the majority had a history of childhood maltreatment. Despite this, we still did not find significant differences in antidepressant response based on trauma load. Additionally, when we conducted separate analyses examining specific types of childhood trauma (i.e., sexual or physical violence) independently, the results remained consistent. Unlike O'Brien et al. [27], a history of childhood sexual or physical abuse did not correlate with improved antidepressant outcomes following ketamine treatment, further supporting that childhood maltreatment, as measured in our study, did not influence ketamine's antidepressant effects.

Another important consideration is that the version of the CTQ used in our study does not assess childhood maltreatment in the form of physical neglect, emotional neglect, or emotional abuse. If individuals in our High Load group were exposed to a wider range of adversities beyond maltreatment, but those in O'Brien et al.'s study had more severe maltreatment exposure (e.g., chronic emotional or physical abuse that was not captured in our measure), this could partially explain why their study found a stronger association between childhood trauma and ketamine response. Additionally, some individuals classified as Low Load in our study may have experienced significant maltreatment that was not captured in our measure. If these unmeasured adversities influence treatment response, our findings could underestimate the impact of childhood maltreatment on ketamine's effectiveness.

These differences in trauma measurement highlight the challenges of defining and categorizing childhood adversity. They also suggest that discrepancies between our findings and those of O'Brien et al. [27], may stem, at least in part, from variations in how childhood trauma was assessed. Future research using more comprehensive trauma assessments will be important for further clarifying the relationship between childhood trauma and ketamine response.

A key strength of this study lies in its real‐world design, enhancing the generalizability of findings. The use of a community outpatient sample reflects real‐world conditions, providing valuable insights into ketamine's effectiveness outside of controlled trial settings. Additionally, our comprehensive assessment of childhood trauma, encompassing both maltreatment and other adversities, offers a nuanced perspective on the role of childhood trauma in treatment outcomes.

Despite these strengths, several limitations must be acknowledged. First, data collection from community outpatient ketamine clinics introduced potential expectancy bias, as participants were aware they were receiving ketamine and paid for treatments out‐of‐pocket or through insurance. The lack of a placebo control group further limits the ability to isolate ketamine's true antidepressant effects from placebo or motivational influences [36]. Second, the retrospective design and reliance on self‐reported trauma histories introduce the potential for recall bias, where participants may have over‐ or under‐reported their childhood trauma experiences [37]. Third, the relatively small sample size reduced the statistical power to detect smaller effects and limited the exploration of baseline characteristics such as psychiatric comorbidity, education level, socioeconomic status (SES), treatment history, or insurance coverage status. Previous research has highlighted the importance of identifying baseline characteristics that may influence response to acute low‐dose ketamine infusions. For example, predictors such as body mass index (BMI), family history of alcohol use disorder, suicide history, neuroimaging markers (e.g., anterior cingulate cortex activity), and cognitive functioning (e.g., processing speed) have been associated with differential response rates to ketamine treatment [38]. We did not collect data on these measures in the present study, but these findings underscore the complexity of ketamine's antidepressant mechanisms and suggest that factors beyond childhood trauma, including metabolic‐inflammatory alterations and cognitive impairment, may play a critical role in shaping treatment outcomes and should be investigated in future studies. Finally, the version of the CTQ used in this study also presents limitations. It lacks established clinical cut‐offs for trauma severity and does not quantify the frequency or chronicity of traumatic events, reducing comparability with studies that employ more detailed versions of the CTQ. This limitation may have led to misclassification of participants or insufficient representation of trauma severity, potentially influencing the findings.

Future research should aim to address these limitations. Larger, placebo‐controlled trials are needed to replicate these findings and determine whether ketamine's effectiveness is sustained over longer periods. Studies should employ more nuanced outcome measures that differentiate childhood maltreatment (e.g., abuse or neglect) from other adverse childhood experiences (e.g., parental divorce), as these may have distinct impacts on treatment response. Additionally, considering the frequency and chronicity of traumatic experiences is crucial, as repeated trauma exposure may more strongly predict treatment outcomes compared to isolated events. Furthermore, investigating the neurobiological mechanisms underlying ketamine's antidepressant effects in individuals with a history of childhood trauma could also offer valuable insights into how trauma influences treatment outcomes. For example, it has been documented that ketamine and other glutamatergic modulators may have anti‐inflammatory effects, possibly contributing to antidepressant outcomes in persons with MDD and trauma [39]. This research could help clarify the pathways through which childhood trauma affects the brain's response to ketamine and identify potential biomarkers for predicting treatment success.

## 5 Conclusion

This study contributes to the growing body of evidence supporting the use of ketamine for TRD and underscores its potential effectiveness in individuals with a history of childhood trauma. Although childhood trauma is a known risk factor for treatment resistance in depression, our findings suggest that ketamine may be an effective treatment option even when other treatment modalities have failed. These results underscore the need for continued research to further elucidate ketamine's role in treating this complex population.

## Author Contributions

**Danica E. Johnson:** conceptualization, methodology, formal analysis, writing – original draft, writing – reviewing and editing. **Nelson B. Rodrigues:** formal analysis, writing – reviewing and editing. **Rodrigo B. Mansur:** supervision, writing – reviewing and editing. **Roger S. McIntyre:** resources, supervision, writing – reviewing and editing. **Joshua D. Rosenblat:** resources, supervision, writing – reviewing and editing.

## Ethics Statement

This study was approved by a community Institutional Review Board (IRB#00000971) and is registered at clinicaltrials.gov under the identifier NCT04209296. This research received no specific grant from any funding agency, commercial or not‐for‐profit sectors.

## Conflicts of Interest

Danica E. Johnson's salary is supported by the Ontario Graduate Scholarship (OGS) and Canadian Institutes of Health Research (CIHR), but there was no direct project funding for the present study. Dr. Joshua D. Rosenblat has received research grant support from the Canadian Institute of Health Research (CIHR), Physician Services Inc. (PSI) Foundation, Labatt Brain Health Network, Brain and Cognition Discovery Foundation (BCDF), Canadian Cancer Society, Canadian Psychiatric Association, Academic Scholars Award, American Psychiatric Association, American Society of Psychopharmacology, University of Toronto, University Health Network Centre for Mental Health, Joseph M. West Family Memorial Fund, Inagene and Timeposters Fellowship and industry funding for speaker/consultation/research fees from iGan, Boehringer Ingelheim, Braxia Health (Canadian Rapid Treatment Centre of Excellence), Braxia Scientific, Janssen, Allergan, Lundbeck, Sunovion and COMPASS. Dr. Roger S. McIntyre has received research grant support from CIHR/GACD/National Natural Science Foundation of China (NSFC) and the Milken Institute; speaker/consultation fees from Lundbeck, Janssen, Alkermes, Neumora Therapeutics, Boehringer Ingelheim, Sage, Biogen, Mitsubishi Tanabe, Purdue, Pfizer, Otsuka, Takeda, Neurocrine, Neurawell, Sunovion, Bausch Health, Axsome, Novo Nordisk, Kris, Sanofi, Eisai, Intra‐Cellular, NewBridge Pharmaceuticals, Viatris, Abbvie and Atai Life Sciences.

## Peer Review

The peer review history for this article is available at https://www.webofscience.com/api/gateway/wos/peer‐review/10.1111/acps.13812.

## Supporting information


**Figure S1.** (A–F) CTQ Questions 1–6. Each line represents the mean QIDS‐SR16 scores at each time point for participants who responded “Yes” (trauma type present) or “No” (trauma type absent) to the respective CTQ question. Error bars represent 95% confidence intervals.

## Data Availability

Due to the confidential nature of the collected data and research ethics requirements, the dataset will not be made publicly available. However, data sharing may be considered on a case‐by‐case basis upon request, subject to a signed data‐sharing agreement and approval from the appropriate ethics board.
