# Supplementary material for: The Influence of Childhood Trauma on the Real‐World Effectiveness of Ketamine in Adults With Treatment‐Resistant Depression
Source: Acta Psychiatr Scand. 2025 Apr 16;152(2):134–45. doi: 10.1111/acps.13812 (PMC12213008; doi:10.1111/acps.13812)
Supplement: Supplementary file 1 — Figure S1. (A–F) CTQ Questions 1–6. Each line represents the mean QIDS‐SR16 scores at each time point for participants who responded “Yes” (trauma type present) or “No” (trauma type absent) to the respective CTQ question. Error bars represent 95% confidence intervals. [file ACPS-152-134-s001.docx]

**Supplementary Material**

| 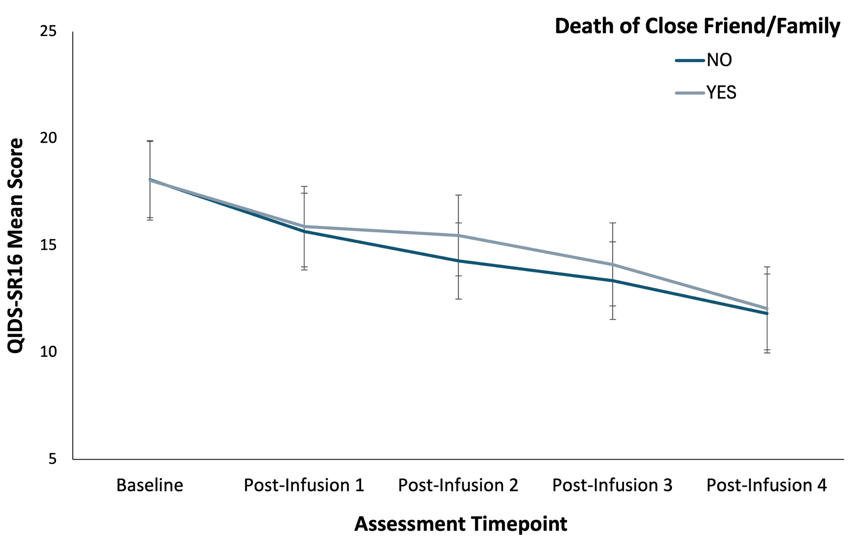  **C** | 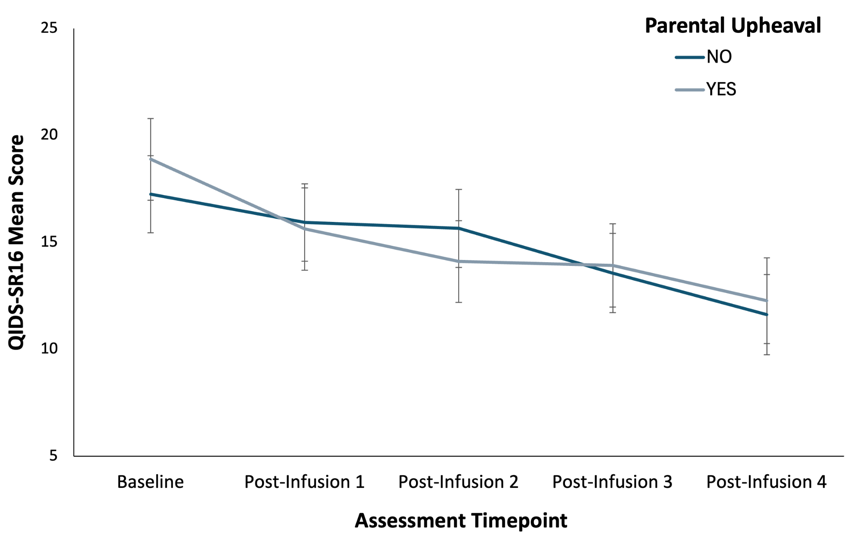  **D**  **B** |
| --- | --- |
| 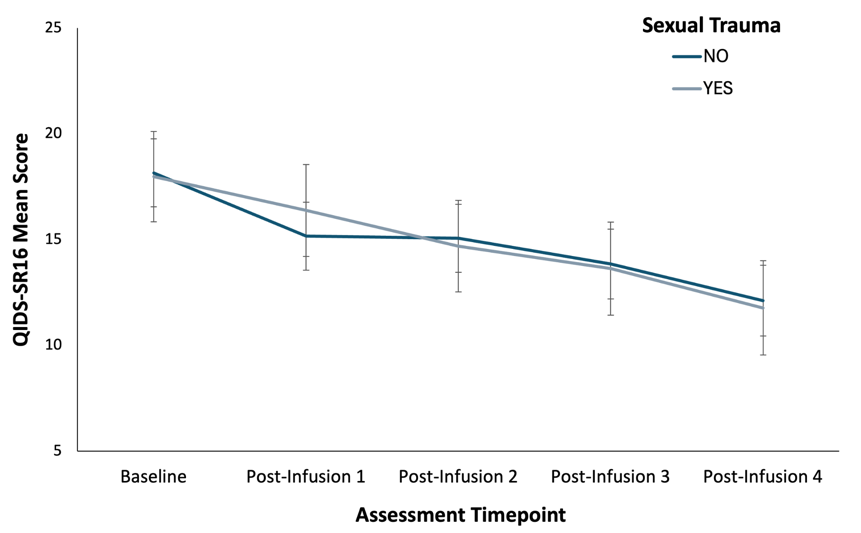  **E** | 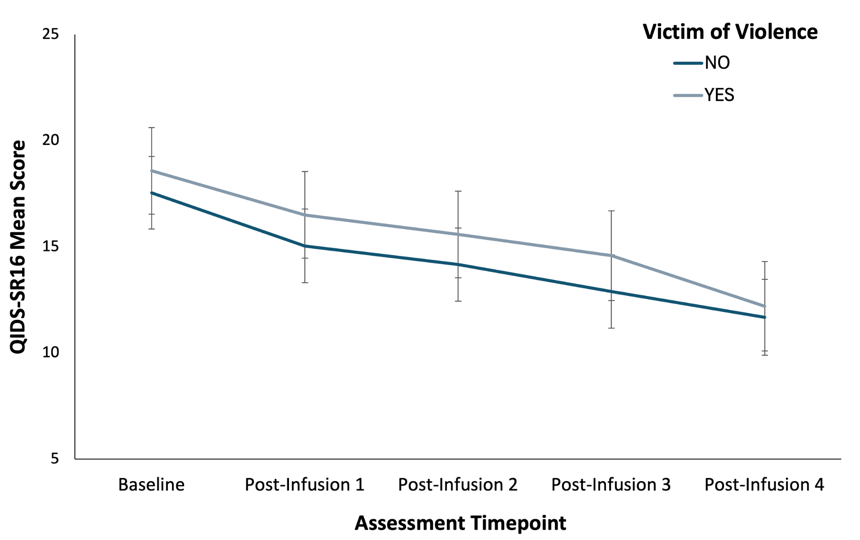  **F** |
| 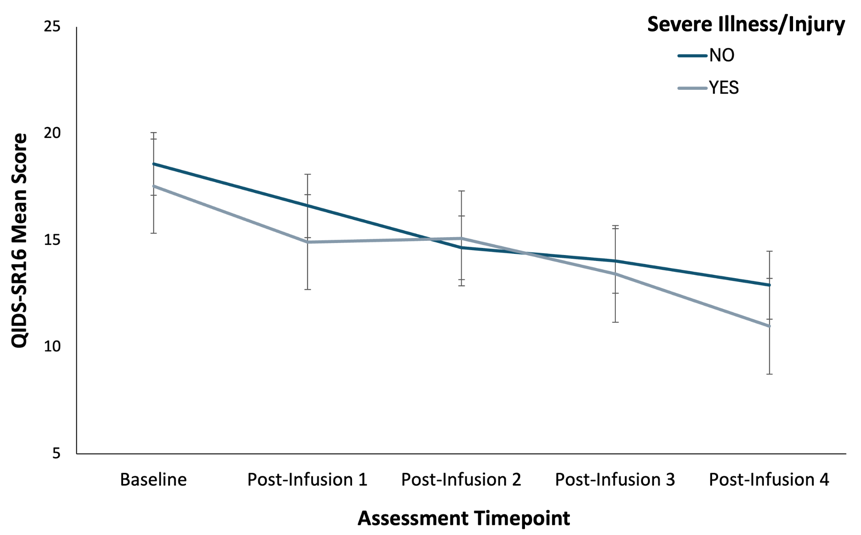 | 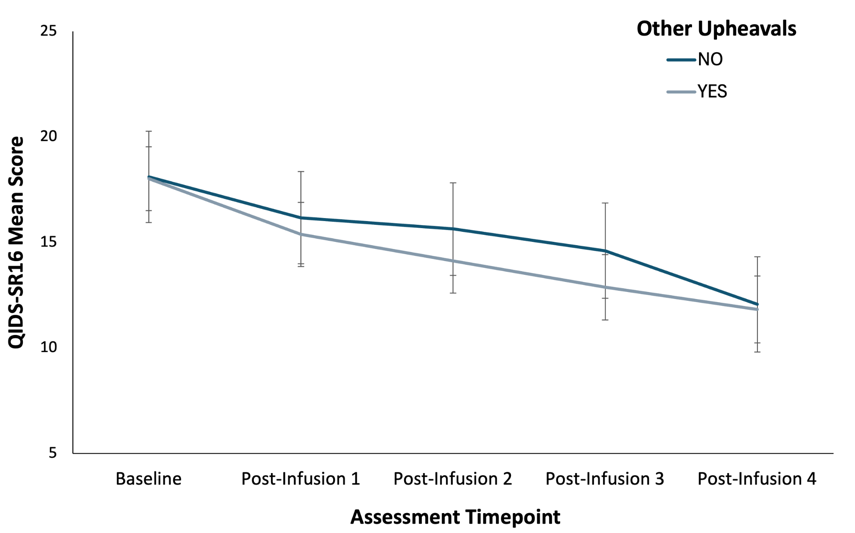 |

**A**

**Supplementary Figure 1.** Panels A–F correspond to CTQ Questions 1–6, respectively. Each line represents the mean QIDS-SR16 scores at each time point for participants who responded 'Yes' (trauma type present) or 'No' (trauma type absent) to the respective CTQ question. Error bars represent 95% confidence intervals.
